# Comparing Survival of Israeli Acute Paralysis Virus Infection among Stocks of U.S. Honey Bees

**DOI:** 10.3390/insects12010060

**Published:** 2021-01-12

**Authors:** Shilpi Bhatia, Saman S. Baral, Carlos Vega Melendez, Esmaeil Amiri, Olav Rueppell

**Affiliations:** 1 Department of Biology, University of North Carolina Greensboro, 321 McIver Street, Greensboro, NC 27403, USA; Sbhatia@aggies.ncat.edu (S.B.); samanbaral@gmail.com (S.S.B.); carlos.vegamelendez@usda.gov (C.V.M.); e_amiri@uncg.edu (E.A.); 2 Department of Applied Science & Technology, North Carolina Agricultural & Technical University, 1601 E Market Street, Greensboro, NC 27411, USA; 3 US Dairy Forage Research Center, USDA-ARS, 1925 Linden Drive, Madison, WI 53706, USA; 4 Department of Biological Sciences, University of Alberta, Edmonton, AB T6G 2E9, Canada

**Keywords:** genetic variation, immunity, pollinator health, selective breeding, apiculture, queen rearing

## Abstract

**Simple Summary:**

Honey bees and other pollinators are threatened by numerous stressors, including virus infections. Currently, no effective treatments are available, stressing the importance of natural defenses. These defenses may be enhanced through selective breeding. This study sought to evaluate the potential for breeding, while also testing a few potential mechanisms of natural immune responses and assessing how widespread viruses are in commercial honey bee queens in the U.S. We identified significant differences in survival of virus infection among and within U.S honey bee stocks, indicating that selective breeding may be able to decrease the virus susceptibility of honey bees. Survival differences may be related to differences in the natural immune system of honey bees and could relate to how much virus stress bees have experienced in the past.

**Abstract:**

Among numerous viruses that infect honey bees (*Apis mellifera*), Israeli acute paralysis virus (IAPV) can be linked to severe honey bee health problems. Breeding for virus resistance may improve honey bee health. To evaluate the potential for this approach, we compared the survival of IAPV infection among stocks from the U.S. We complemented the survival analysis with a survey of existing viruses in these stocks and assessing constitutive and induced expression of immune genes. Worker offspring from selected queens in a common apiary were inoculated with IAPV by topical applications after emergence to assess subsequent survival. Differences among stocks were small compared to variation within stocks, indicating the potential for improving honey bee survival of virus infections in all stocks. A positive relation between worker survival and virus load among stocks further suggested that honey bees may be able to adapt to better cope with viruses, while our molecular studies indicate that toll-6 may be related to survival differences among virus-infected worker bees. Together, these findings highlight the importance of viruses in queen breeding operations and provide a promising starting point for the quest to improve honey bee health by selectively breeding stock to be better able to survive virus infections.

## 1. Introduction

The Western honey bee (*Apis mellifera*) is the world’s most important managed pollinator for agricultural crops and also pollinates a number of natural flowers [1]. Over the past years, a number of potentially interacting threats to honey bee health have been identified, including pesticides, nutritional deficits, management stress, as well as pests and pathogens [2,3,4,5,6,7,8,9]. Among the biological stressors, the parasitic *Varroa* mite and a range of viruses have particularly profound impacts on individual and colony health [10,11,12,13]. Over 30 viruses have been reported from honey bees so far with pathological effects ranging from acute mortality of individuals and colony collapse to covert asymptomatic infections [8,12,13,14,15,16,17]. One of the more notorious viruses is Israeli acute paralysis virus (IAPV), which has been associated with colony collapse [18] and can affect every member of honey bee colonies with a widespread internal distribution [19]. It can cause bee death when injected at sufficient doses [20]. However, it also can cause sublethal infections [21,22] and persists asymptomatically at low quantities in honey populations, although it is far from ubiquitous [18,19]. Even though RNA interference (RNAi)-based vaccines against IAPV and other viruses are under development [23,24], selectively improving the existing honey bee immune defenses presents a practical and sustainable solution for improving honey bee health [25].

Honey bees rely on a combination of societal and individual immunity [26]. Social immunity involves collective defenses against diseases [27] and range from social transfers of immune system elicitors [28] and social apoptosis [29] to behavioral adjustments [22] and active defenses [30,31], to nest construction [32] and colony organization [33,34]. At the individual level, honey bees rely on a combination of cellular and humoral immunity, similar to other insects [35]. They possesses all major immune pathways, including almost all members of the Toll, Imd, JNK, Tor, and Jak-STAT pathways that activate the antimicrobial peptides abaecin, hymenoptaecin, apidaecin, and defensins [36]. Particularly important for honey bee viral defenses is their functional RNAi pathway, which has been directly linked to a functional antiviral response [37,38].

Although the global expansion of *A. mellifera* is due to its cultivation by humans, the species is highly adaptable, and several distinct subspecies have naturally evolved across its wide geographic distribution [39]. Through a combination of targeted selection/breeding and less deliberate introduction of genetic material from several of its naturally evolved subspecies into the managed apicultural population of North America, considerable genetic variation is represented in these commercial honey bees [40,41]. Genetic variation within colonies is promoting disease resistance [42], potentially by enhancing division of labor and thus social immunity. However, the standing genetic variation within populations may also directly relate to differences in disease susceptibility if certain genotypes confer an advantage in disease resistance or tolerance. Several populations survive *Varroa* infestation by more efficient hygienic behavior [43,44] but some survival benefits may also be due to virus tolerance [45,46].

Evidence for genetic variation in *Varroa* resistance is strong [47,48,49] and specific genome regions that influence various aspects of this compound trait have been identified [31,50,51]. As a result, multiple breeding lines have successfully been established and propagated to be disseminated to the apicultural industry in North America, including Minnesota Hygienic bees [30,52], the USDA-bred POL-line derived from Varroa-Sensitive Hygiene bees [53], and “Russian” honeybees [54]. In contrast, genetic variation for the interactions between viruses and their honey bee hosts is less well established, even though such knowledge constitutes an essential foundation for sustainable breeding efforts against viral diseases. Some experimental support for selectable genetic variation in virus resistance exists for Chronic bee paralysis virus [55], variation between colonies in IAPV titer dynamics exists [20], and the activity of immune genes is also heritable [56,57]. However, more data are needed to evaluate the potential for selectively breeding for virus resistance in honey bees.

In this study, we assess variation in survival of IAPV infection in honey bee workers of different stocks under controlled laboratory conditions. We hypothesize that genetic variation among honey bee stocks causes differences in virus susceptibility and predict that IAPV inoculations of different genetic stocks under identical conditions lead to significant differences in survival among stocks. We complement the test of this prediction with molecular surveys of the virus content of the investigated stock to study a potential relation between resident viruses and IAPV susceptibility. Furthermore, we investigate the constitutive and induced expression levels of several immune genes to evaluate them as potential explanations for naturally occurring differences in virus susceptibility of honey bees.

## 2. Materials and Methods

### 2.1. Honey Bee Stocks

Ten naturally open-mated queens from six different stocks were obtained in the spring of 2017. While the exact sources cannot be named here due to privacy concerns, they generally represented Minnesota Hygienic (MHY), POL-line (POL), Russian (RUS) bees, as well as “Italians” from California (ITC) and Georgia (ITG) and “Carniolans” from Hawaii (CAH). These stocks were selected to represent a wide variety of commercially available American stocks [41]. While the former three represent breeding efforts against *Varroa*, systematic comparisons of these stocks in other traits, such as vitality or productivity, have not yet been conducted.

### 2.2. Experimental Treatment, Survival Analysis, and Sampling

Upon arrival, four queens from each source were directly sacrificed and frozen at −80 °C. Prior to dissections, these queens were soaked at −20 °C in RNAlaterICE^®^ (Applied Biosystems, Foster City, CA, USA) overnight to allow for tissue collection without RNA degradation for subsequent analyses. Each queen was pinned onto a sterile dissection dish and the head, thorax, gut, spermatheca, and ovaries were isolated and frozen until further processing for screening of virus titers (see below). While the queens may differ in virus content from their workers [58], vertical transmission has been demonstrated for several viruses (e.g., Deformed wing virus—DWV [59]), and queens are most widely distributed throughout the U.S. apicultural industry.

The remaining six queens from each source were marked on their thorax with different colors to represent their sources and introduced into 3-frame mating nucs containing 500–700 worker bees from common source colonies in an isolated apiary maintained at the University of North Carolina at Greensboro, NC. Colonies were inspected every other day and received sugar patties when needed as supplemental food and newly emerged workers were added to maintain colony size. No Varroa or other disease treatments were necessary during the experimental period. Upon completion of the 21-day brood cycle, frames of emerging brood from each colony were collected and transferred into an emergence incubator (33 °C, 60% RH). Approximately 100–500 newly emerged workers were kept on their frames until 3–5 days of age and then subjected to IAPV inoculation or a sham treatment. Around ten untreated bees were frozen at −80 °C for each trial as a pre-treatment reference sample.

After anaesthetization with CO_2_ for 10–12 s, the thoracic hairs were shaved off and individuals either inoculated by applying an aqueous solution of purified IAPV particles onto their thorax [21] or received a water solution as sham treatment. Experimental treatment groups were separated into three subgroups of 15 individuals that were housed in separate single-use plastic cups [60]. Cups of treatment (IAPV-inoculated) and sham (water-treated) groups were maintained separately in an incubation chamber (30 °C, 45% RH) for survival analysis. We used a topical IAPV infection model previously established in our lab instead of direct injection because we found topical applications resulting in a more graded response [21]. Furthermore, we found it more efficient than feeding and less traumatic than injection. Bees were monitored for survival starting 8 h post infection at three time points per day (6 am, 2 pm and 10 pm) until all bees in the treatment cups of a particular trial had died. At that time, data recording for the corresponding control cups from the same trial was also stopped. In cases when treatment groups were still alive 2 weeks (=336 h) after infection, data collection was invariably stopped and any bees alive were frozen at −80 °C. All dead bees were collected during each inspection time point and frozen at −80 °C for subsequent molecular analysis. Depending on brood availability, one to five trials per colony (17 colonies in total) were performed. Each trial represented three cups of 15 IAPV-inoculated and three cups of 15 sham-treated workers, but in two instances the number of emerging workers only sufficed to set up one cup of each, sham and IAPV treatment. IAPV inoculum used in this study was purified, quantified, and prepared (in PBS) as described in [20]. The IAPV inoculum contained 5.24 × 10^7^ genome copies of IAPV per microliter, less than 10^3^ copies of DWV and Sacbrood virus (SBV), and no measurable Acute bee paralysis virus (ABPV) or Black queen cell virus (BQCV).

Data for each colony and treatment were pooled across cups and trials after excluding individual deaths in the first eight-hour interval (because these deaths were likely caused by experimental manipulations) and cups that exhibited >50% mortality in any given time interval (because such unusually high mortality was likely due to husbandry problems in that particular cup). To assess the overall effect of IAPV-inoculation vs. sham treatment, a survival comparison between the two groups across all colonies was performed with a log-rank test (Kaplan–Meier module in SPSS v.20 (IBM). After survival of the sham treatment proved significantly different among colonies but not stocks, we continued our survival analysis with absolute survival times of individuals after IAPV inoculation ($S_{IAPV}$) and relative survival ($S_{rel}$), using the average survival time of sham-treated workers of a colony (${\bar{S}}_{Sham}$) to correct each individual survival time of IAPV-inoculated workers according to $S_{rel}=S_{IAPV}-{\bar{S}}_{Sham}+336$. The theoretical maximum survival of 336 was added to make all *S_rel_* values positive. Absolute and relative survival was compared among stocks and colonies with a hierarchical (2-level) survival analysis using Cox proportional hazard models with mixed effects in “R”, using the “survival” package v. 3.1–12.

### 2.3. RNA Extraction and cDNA Synthesis

Separate queen body parts and tissues, as well as whole worker bees from the inoculation assays were homogenized using a micro-pestle in a 1.5 mL centrifuge tube, followed by a total RNA extraction with an established TRIzol™ (Invitrogen, Carlsbad, CA, USA) protocol [61]. The concentration and purity of the extracted RNA samples were measured using a Nanodrop ND-1000 spectrophotometer (Thermo Scientific, Wilmington, DE, USA), and total RNA concentration was adjusted to 20 ng/µL in molecular grade water (Fisher Scientific, Fair Lawn, NJ, USA). Thereafter, cDNA was synthesized for each sample using the High-Capacity cDNA Reverse-Transcription Kit (Applied Biosystems, Foster City, CA, USA). Ten microliters of the RNA template (20 ng/µL) were added to 10 µL of the provided cDNA master mix, followed by an incubation period as recommended by the manufacturer: 10 min at 25 °C, 120 min at 37 °C, and 5 min at 85 °C. The resulting cDNA solution was then diluted 10-fold in molecular grade water and stored at −80 °C to serve as the template in subsequent qPCR-based analysis of immune gene expression and virus quantification.

### 2.4. Quantification of Viruses

Viruses were quantified in all samples with successful cDNA product from the initially surveyed queens. In addition, viruses were quantified in the pretreatment worker groups. qRT-PCR was performed using primers specific for the following viruses: Black queen cell virus (BQCV), Deformed wing virus (separately: DWV-A, DWV-B, and DWV-C), Israeli acute paralysis virus (IAPV), and Kashmir bee virus (KBV), Sacbrood virus (SBV), Acute bee paralysis virus (ABPV), and Chronic bee paralysis virus (CBPV) (see Table S1A for a listing of the primers and associated references). To assess the validity of our experimental inoculations, IAPV was also quantified in select worker samples that were collected in the process of dying after 2–3 days from the IAPV-inoculated groups during the survival experiment. β-actin and RPS5 were used as the internal reference genes [62]. In addition, RNase-free water was added as template for a No Target Control (NTC), and a No Reverse Transcriptase (NRT) control served as an additional negative control. The samples were run in a 96-well plate in duplicates. The thermal cycling conditions using a StepOnePlus™ (Applied Biosystems, Foster City, CA, USA) were set at 10 min at 95 °C, followed by 40 cycles consisting of a denaturing stage at 95 °C for 15 s and an annealing/extension stage at 60 °C for 1 min. Fluorescence measurements were taken at the end of each cycle. This procedure was followed by a final melt-curve dissociation analysis to confirm the specificity of the products. Samples were deemed positive for a target if their melting temperature was similar to the melting temperature of the positive controls and a C_t_ value of 35 or lower was recorded.

Virus content of the queens was compared for each tissue and virus separately by ANOVA. In addition, overall virus load of each stock was compared across all tissues and viruses in a separate ANOVA. Specific and overall virus loads were correlated to the average survival times of IAPV infected workers across the six stocks to test whether average survival was related to the general viral exposure of these stocks. The quantities of each virus in the pretreatment worker groups were independently correlated to the worker survival of the corresponding colonies to test whether differences in viruses before IAPV inoculation were correlated with survival differences among colonies. Finally, IAPV quantities were compared between IAPV-inoculated and noninoculated workers to confirm the efficiency of our experimental treatments.

### 2.5. Quantification of Immune Genes

To assess whether the constitutive or virus-induced expression of select immune genes was related to differences in worker survival of IAPV among colonies, samples from nine colonies were selected for gene expression analysis based on their survival of IAPV inoculation. Frozen worker bees from these colonies that were collected before (pretreatment control) and 2–3 days after IAPV-inoculations (experimental group) were included. The following antimicrobial peptides (AMPs) and other immune genes were selected as targets based on previous studies: dicer-like, argonaute (AGO-2), toll, hopscotch, apidaecin, and hymenoptaecin (see Table S1B for a listing of the specific primers used and the associated references). qPCRs were performed as described above, including positive and negative controls and reference genes on every plate. The constitutive expression of each immune gene was determined as ∆CT by subtracting the CT value of the average of reference genes (see above) from the CT value of the target [63]. To quantify relative induction of the same genes, ∆∆CT was calculated by computing the difference between the ∆CT of experimental (post-treatment) workers from the ∆CT of the pretreatment group (∆CT experimental−∆CT pretreat) [64]. For each gene, the average pretreatment ∆CT and the average ∆∆CT of each colony were correlated with the survival of IAPV-inoculated workers relative to control survival using Spearman’s rank correlation analysis to test whether colony differences in candidate immunity mechanisms could explain survival differences.

## 3. Results

### 3.1. Virus Survey of Stocks

The RT-qPCR surveys of thorax, gut, and ovaries of queens from the six stocks revealed the presence of numerous viruses. The overall virus prevalence (all viruses) across all body parts was significantly different among stocks (Χ^2^ = 16.6, df = 5, *p* = 0.005). Overall virus prevalence was significantly different among stocks only in the gut (Χ^2^ = 13.7, df = 4, *p* = 0.008) but not in the thorax (Χ^2^ = 7.6, df = 5, *p* = 0.181) or ovary (Χ^2^ = 5.3, df = 4, *p* = 0.261).

**Figure 1:**
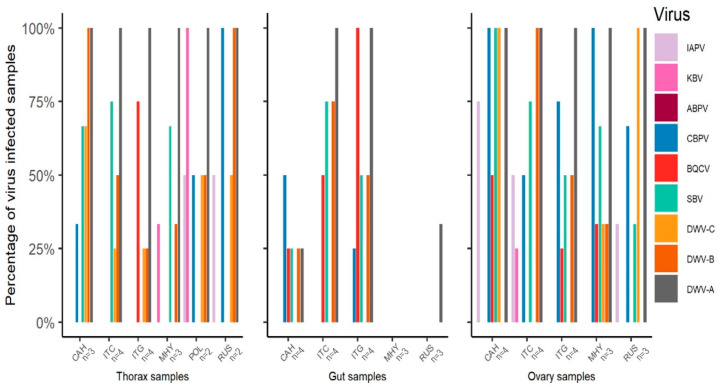
Percentage of samples testing positive for the presence of different viruses in different body parts (thorax, gut, and ovaries). Newly shipped queens from different stocks (ITC = “Italians” from California, ITG = “Italians” from Georgia, CAH = “Carniolans” from Hawaii, MHY = Minnesota “Hygienics”, POL = POL-line, and RUS = “Russians”) were tested for the presence of Israeli acute paralysis virus (IAPV), Kashmir bee virus (KBV), Acute bee paralysis virus (ABPV), Chronic bee paralysis virus (CBPV), Black queen cell virus (BQCV), Sacbrood virus (SBV), Deformed wing virus DWV-A, DWV-B, and DWV-C via RT-PCR. Sample sizes are indicated below the *x*-axis.

Analyzed separately (Figure 1), KBV (Χ^2^ = 16.6, df = 5, *p* = 0.005), CBPV (Χ^2^ = 19.5, df = 5, *p* = 0.002), BQCV (Χ^2^ = 21.8, df = 5, *p* = 0.001), DWV-B (Χ^2^ = 19.2, df = 5, *p* = 0.002), and DWV-A (Χ^2^ = 11.5, df = 5, *p* = 0.042) were significantly different among stocks across all tissues. When analyzing the quantitative viral loads of each tissue separately, significant differences were found in the thorax for KBV (F_(5,12)_ = 3.5, *p* = 0.035), CBPV (F_(5,12)_ = 20.7, *p* < 0.001), and BQCV (F_(5,12)_ = 4.7, *p* = 0.013), in the gut for BQCV (F_(4,13)_ = 11.8, *p* < 0.001), DWV-A (F_(4,13)_ = 7.8, *p* = 0.002), DWV-B (F_(4,13)_ = 3.4, *p* = 0.041), and DWV-C (F_(4,13)_ = 3.4, *p* = 0.040), and in the ovary for DWV-B (F_(4,13)_ = 7.7, *p* = 0.002) and DWV-C (F_(4,13)_ = 13.7, *p* < 0.001).

### 3.2. Variation in IAPV-Inoculated Worker Survival among Honey Bee Colonies and Stocks

**Figure 2:**
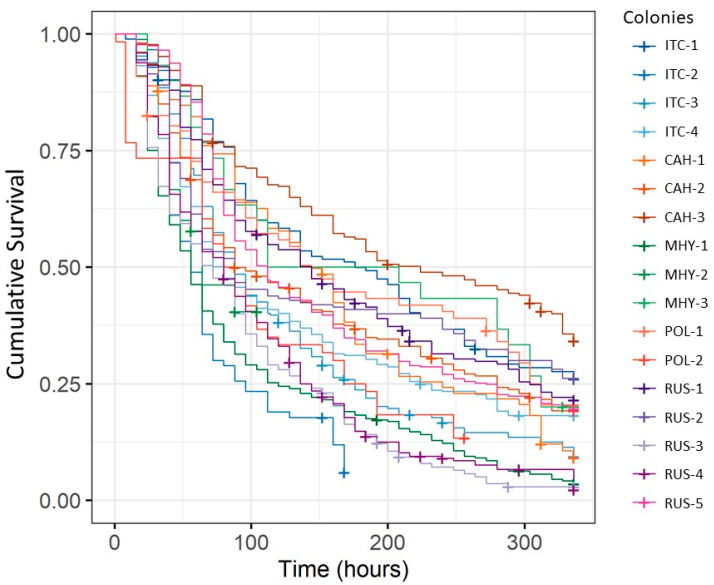
Survival of IAPV-inoculated worker honey bees from colonies of different stocks. Each line represents survival time of workers from one colony of a particular stock (ITC = “Italians” from California, CAH = “Carniolans” from Hawaii, MHY = Minnesota “Hygienics”, POL = POL-line, and RUS = “Russians”). Average survival, as well as survival dynamics, differed profoundly among colonies. “+” indicates censored data.

Altogether, the survival of 2027 control-treated and 2030 IAPV-inoculated workers was assessed to study differences between stocks and colonies. IAPV-inoculated worker survival was significantly different among stocks (Χ^2^ = 42.7, df = 4, *p* < 0.001; Table 1) but differences among colonies within stocks were more pronounced (Χ^2^ = 189, df = 16, *p* < 0.001). Relative survival was also significantly different among stocks (Χ^2^ = 75.9, df = 4, *p* < 0.001) but the differences were not the same (Table 2) and colony differences were again much more pronounced (Χ^2^ = 541, df = 16, *p* < 0.001; Figure 2). IAPV survival but not relative survival of the stocks was positively correlated to total virus loads experienced by these stocks (expressed as the negative of the average ∆CT), although our small sample size precluded a precise evaluation (thorax: R_P_ = 0.45, *n* = 5, *p* = 0.448; gut: R_P_ = 0.99, *n* = 4, *p* = 0.002; ovary: R_P_ = 0.76, *n* = 4, *p* = 0.243).

### 3.3. Correlation of IAPV-Inoculated Worker Survival with Immune Gene Expression

**Figure 3:**
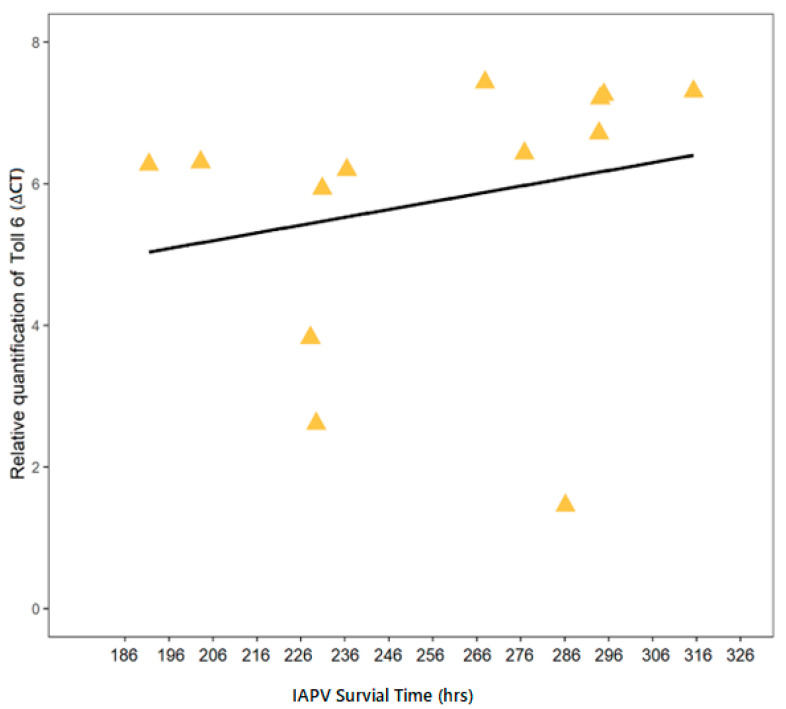
The constitutive expression of the immune gene toll-6 was related to IAPV survival across colonies. Average expression of toll-6 (measured in pretreatment samples as ∆CT relative to two reference genes) was positively correlated (Rs = 0.56, *n* = 13, *p* = 0.0499) to average IAPV survival across colonies. Yellow triangles indicate individual data points and the black line describes the linear trend.

To test whether the pre-experimental expression levels of several immune genes were correlated with worker survival of IAPV exposure, their average ∆CT values were correlated with the average survival of IAPV-inoculated workers (relative to control survival) of each colony. Toll-6 expression showed a significant correlation to IAPV survival across colonies (Rs = 0.56, *n* = 13, *p* = 0.0499; Figure 3). Toll-6 also exhibited the strongest correlation between IAPV survival and induced expression, measured as immune gene expression after inoculation relative to pre-experimental expression levels (∆∆CT). However, this relation was not significant (Rs = −0.57, *n* = 12, *p* = 0.055). IAPV survival was not significantly correlated to constitutive or induced expression of any other candidate gene.

### 3.4. Relation of IAPV-Inoculated Worker Survival to Virus Titers

No evidence of CBPV, KBV, and DWV-C was found in the pretreatment workers. Among the remaining viruses, relative survival of IAPV was negatively correlated to the quantity of BQCV in pretreated workers across colonies (R_S_ = −0.87, *n* = 13, *p* < 0.001). However, when the two components of “relative survival” were correlated to BQCV quantity, it became clear that this negative relation was mainly due to a positive correlation of control survival (R_S_ = 0.66, *n* = 13, *p* = 0.016) and not an effect of BQCV on survival of IAPV-inoculated workers (R_S_ = 0.16, *n* = 13, *p* = 0.604). None of the other viruses (IAPV, SBV, ABPV, DWV-A, and DWV-B) found in the pretreated workers were significantly related to survival differences among colonies. While relative survival was not significantly correlated to the change in IAPV titers {∆∆CT(IAPV)} due to inoculation (R_S_ = 0.43, *n* = 13, *p* = 0.161), survival of the control workers (R_S_ = 0.65, *n* = 13, *p* = 0.026) and of inoculated workers (R_S_ = 0.70, *n* = 13, *p* = 0.014) was significantly correlated with ∆∆CT(IAPV).

## 4. Discussion

Our study was designed to evaluate differences in survival of virus infection among worker honey bees from different stocks and test for a relation between survival and viral loads in the respective source populations. We found significant differences in viral loads among queens tested from different stocks. We also identified some survival differences of IAPV infection among stocks, although variability within stocks was much greater. The survival differences among stocks and among colonies within stocks might indicate variation in virus resistance and thus provide a basis for selective breeding to enhance virus resistance in all stocks. Measures of candidate gene expression and other viruses indicated that survival differences after IAPV inoculation may be in part explained by Toll-6, an important immune gene, but more mechanistic follow-up studies are necessary to elucidate the genetic basis of virus resistance in honey bees.

Our survey of young queens that we acquired from different sources was limited by a relatively small number of successfully evaluated individuals and tissues. This limitation prompted us to evaluate the prevalence of viruses across all samples as measure of pathogen pressure in the source populations, even though different tissues of the same individual cannot be considered strictly independent of each other [65]. When analyzed alone, only differences in overall viral prevalence of the gut remained significantly different among stocks. However, significant quantitative differences for single viruses were common even when just individual tissues were compared. Overall, our samples revealed surprisingly high virus prevalence: across viruses and tissues, between 26 and 72% of samples from a given stock tested positive. While the widespread detection of DWV and BQCV was expected, the common occurrence of IAPV and CBPV was not [12,66,67]. Potentially, our results reflect peculiarities of queen breeding operations, which are typically stationary and intensively managed with common exchanges of potentially infectious material among hives. Even though varroa is an uncommon vector of viruses to queens, our results suggest that a range of viruses can efficiently infect queens [16]. Another peculiarity of the virus survey was the lower viral prevalence in the gut than in the other two investigated tissues, contrasting with previous studies [19,68,69]. Amplification of the reference genes in the gut samples argue against technical problems with the gut RNA extraction or amplification. Thus, our results may reflect a reduction of gut viruses during the queens’ relative isolation and nutrition in their shipping containers.

Importantly, the virus loads experienced by the different stocks in our study correlated with the differences in IAPV survival. Although our analysis was severely hampered by small sample size, these results suggest that honey bees from high virus environments may have evolved mechanisms to ensure better survival of an acute virus infection. If confirmed, these findings suggest that natural selection is already operating to improve the health of honey bees [70], at least among the different queen breeding operations sampled here. At minimum, our results are in strong contrast to the notion that virus load is higher in some queen breeding operations because their stocks are more susceptible to viruses.

Large sample sizes are required for comparative survival analysis and we measured thousands of individuals. However, the number of individual colonies per stock was limited by absconding of experimental hives or insufficient brood production. Originally planning to analyze the survival of IAPV inoculated workers relative to survival of control workers from each colony, we focused our analyses on the absolute data on IAPV-inoculated worker survival because we found unanticipated differences in survival of sham-treated workers among colonies. To a large extent, these differences could be attributed to a pre-existing infection with BQCV that correlated with survival of control-treated but not of IAPV-inoculated workers. This strong correlation of caged survival with pre-existing BQCV titers is noteworthy because BQCV usually is reported as not harmful to adult workers [12,71].

In the absence of significant effects of resident viruses and other confounding factors, our IAPV-inoculated worker survival differences among and within stocks can be interpreted as genetic differences in IAPV susceptibility, which has also been reported in the egg stage [25]. We chose 3–5 day-old workers to conduct our experiments in workers with a fully-developed immune system because newly-emerged individuals have been shown to be particularly susceptible to disease [72]. We used a common-garden design, establishing all queens in a common apiary under identical conditions. However, colony development and brood production were not identical, which resulted in unequal sample sizes and testing times that were spread over multiple weeks. In general, we were unable to find significant time trends, but we cannot exclude the possibility that differences are at least in part due to variation in colony size or date of testing.

We cannot exclude the possibility that topical applications resulted in some variability of IAPV entering the bees’ body but IAPV titers of inoculated bees were consistently high across all colonies. Additionally, DWV titers were assessed in IAPV-inoculated samples to test for an indirect effect of the inoculation on DWV because previous results indicated that wounding trauma can increase DWV titers [61]. However, the effect of the topical IAPV inoculation on the three DWV variants was highly variable among colonies with no clear overall effect.

Our evaluation of six candidate immune genes suggested that the toll-6 pathway might play a role in the identified differences in IAPV susceptibility: Constitutive expression and expression changes were correlated with IAPV-inoculated worker survival across colonies. However, the expression of toll-6 before inoculation was negatively related to survival, contrary to our expectation. Higher constitutive toll-6 expression could have been related to lower survival because it may have indicated an undetected immune challenge prior to infection. Meanwhile, the stronger induction from low initial expression levels to relatively high levels after IAPV-inoculation was related to higher survival. These findings have to be interpreted cautiously due to our small sample sizes, but toll-signaling has been generally implicated in honey bee responses to viral infection [36], although its upregulation in response to IAPV infection is unclear [19,73]. The absence of other correlations, particularly the predicted functional relation between dicer-like and IAPV resistance [38,74] may be due to an absence of sufficient genetic variation among the studied colonies in addition to the fact that our correlations at the colony level ignore patrilineal and other genetic variation that exists within colonies.

## 5. Conclusions

We report standing genetic variation in virus susceptibility among and within different stocks in the US and tentatively relate this variation to possible explanations, such as toll-6 expression and the viral load of the source populations. Additionally, our findings indicate that viruses may be quite common in queens without clear symptoms. Thus, commercial distribution of queens constitutes an effective transmission pathway for viruses to reach new operations at a large spatial scale. We suggest that queen breeders may want to include virus screening into their management and breeding practices. Although our bioassays using individual inoculation are labor-intensive, new methods could be developed to assess virus-susceptibility more efficiently.

## Figures and Tables

**Table 1:** Hazard ratio (±S.E.) for worker survival of IAPV inoculation for the stock on the top of each column relative to the stock on the left of each row. Hazard ratio refers to the ratio of probability of death, thus values above 1 indicate a higher probability of death in workers of the stock indicated above columns compared to the stock on the left. Significant differences based on uncorrected significant thresholds are typed in bold-face.

	CAH	MHY	POL	RUS
ITC	0.91 ± 0.15 *p* = 0.518	1.14 ± 0.31 *p* = 0.681	0.87 ± 0.17 *p* = 0.416	**0.64 ± 0.14 *p* = 0.002**
CAH		1.25 ± 0.30 *p* = 0.459	**0.61 ± 0.17 *p* = 0.004**	**0.71 ± 0.12 *p* = 0.004**
MHY			**0.49 ± 0.32 *p* = 0.026**	0.56 ± 0.30 *p* = 0.057
POL				**1.62 ± 0.20 *p* = 0.015**

**Table 2:** Hazard ratio (±S.E.) for worker survival of IAPV inoculation relative to survival of sham-treated workers for the stock on the top of each column relative to the stock on the left of each row. Significant differences based on uncorrected significant thresholds are typed in bold-face.

	ITC	CAH	MHY	POL
RUS	**1.77 ± 0.14 *p* < 0.001**	0.99 ± 0.12 *p* = 0.945	**2.29 ± 0.30 *p* = 0.006**	**0.61 ± 0.17 *p* = 0.004**
POL	1.40 ± 0.22 *p* = 0.119	0.78 ± 0.20 *p* = 0.233	1.81 ± 0.34 *p* = 0.081	
MHY	0.77 ± 0.31 *p* = 0.409	**0.43 ± 0.30 *p* = 0.006**		
CAH	**1.79 ± 0.15 *p* < 0.001**			

## Data Availability

Data are available from the first author upon request.

## Supplementary Materials

The following are available online at https://www.mdpi.com/2075-4450/12/1/60/s1. Table S1. Primer sequences for immune genes. Table S2. Primer sequences for viruses.
