# Supplementary material for: Comparing Survival of Israeli Acute Paralysis Virus Infection among Stocks of U.S. Honey Bees
Source: Insects. 2021 Jan 12;12(1):60. doi: 10.3390/insects12010060 (PMC7827508; doi:10.3390/insects12010060)
Supplement: Supplementary file 1 [file insects-12-00060-s001.pdf]

insects MDPI

Supplementary Table S1: The forward and reverse primer sequences of immune genes used for RT-qPCR. The primers used in the study were synthesized by MWG Eurofins.

Gene name	Reverse Primer Sequence	Forward Primer Sequence	References
Actin	GAAGCAAGAATTGACCCACCAA	CCTAGCACCATCCACCATGAA	**Galbraith et al. 2015**
RPS5	TAACGTCCAGCAGAATGTGGTA	AATTATTTGGTCGCTGGAATTG	**Brito, McHale, and Oldroyd 2010**
Toll-6	GACAGGTCGAACGTCTCCAG	TCCGAGGCGTCAACAGGAATCGACC	**Galbraith et al. 2015**
Dicer-Like	TCTCCACTAAGTGCTGCACAA	CCAACAGGAGCTGGAAAAAC	**Galbraith et al. 2015**
Argonaute-2	TTGCGGTGAACTTTGTTGTT	TCAACAGCAGCAATCGGATA	**Galbraith et al. 2015**
Apidaecin	TTTCACGTGCTTCATATTCTTCA	TAGTCGCGGTATTTGGGAAT	**Evans et al. 2006**
Hymenoptaecin	GCGTCTCCTGTCATTCCATT	CTCTTCTGTGCCGTTGCATA	**Evans et.al 2006**
hopscotch (hop)	AACCTCCAAATCGCTCTGTG	TTGTGCTCCTGAAAATGCTG	**Chen et al.2014**

Supplementary Table S2: List of virus-specific primers used.

Target	Reverse Primer sequence	Forward Primer sequence	References
DWV-TYPE A	CTCATTAACTGTGTCGTTGAT	TACTAGTGCTGGTTTTCCTTT	**Kevill et al, 2017**
DWV-TYPE B	CTCATTAACTGAGTTGTTGTC	TACTAGTGCTGGTTTTCCTTT	**Kevill et al., 2017**
DWV-TYPE C	ATAAGTTGCGTGGTTGAC	TACTAGTGCTGGTTTTCCTTT	**Kevill et al., 2017**
IAPV	CTGAATAATACTGTGCGTATC	CCATGCCTGGCGATTCAC	**De Miranda et al., 2010**
ABVP	GCTCCTATTGCTCGGTTTTTCGGT	TTATGTGTCCAGAGACTGTATCCA	**Chen et al., 2006**
BQCV	GCAACAAGAAGAAACGTAAACCAC	TGGTCAGCTCCCACTACCTTAAAC	**Chen et al., 2006**
CBPV	ACTACTAGAAACTCGTCGCTTCG	CGCAAGTACGCCTTGATAAAGAAC	**Blanchard et al., 2007**
KBV	TGTGGGTTGGCTATGAGTCA	GATGAACGTCGACCTATTGA	**Stoltz et al., 1995**
SBV	ACACTGCGCGTCTAACATTCC	AACGTCCACTACACCGAAATGTC	**Blanchard et al., 2014**
